# 168 million years old “marine lice” and the evolution of parasitism within isopods

**DOI:** 10.1186/s12862-017-0915-1

**Published:** 2017-03-09

**Authors:** Christina Nagler, Matúš Hyžný, Joachim T. Haug

**Affiliations:** 1 Functional morphology group, Department of Biology II, Ludwig-Maximilians-University, Großhaderner Strasse 2, 82152 Planegg-Martinsried, Germany; 2 Department of Geology and Palaeontology, Faculty of Natural Sciences, Comenius University, Mlynská dolina, Ilkovičova 6, 84215 Bratislava, Slovakia; 3 Geological-Paleontological Department, Natural History Museum Vienna, Burgring 7, A-1010 Vienna, Austria; 4 GeoBio-Center, Richard-Wagner Strasse 10, 80333 Munich, Germany

**Keywords:** Cymothoida, Isopoda, *Urda*, Fossil life habits, Evolution, Fossil parasitism

## Abstract

**Background:**

Isopods (woodlice, slaters and their relatives) are common crustaceans and abundant in numerous habitats. They employ a variety of lifestyles including free-living scavengers and predators but also obligate parasites. This modern-day variability of lifestyles is not reflected in isopod fossils so far, mostly as the life habits of many fossil isopods are still unclear. A rather common group of fossil isopods is *Urda* (190-100 million years). Although some of the specimens of different species of *Urda* are considered well preserved, crucial characters for the interpretation of their lifestyle (and also of their phylogenetic position), have so far not been accessible.

**Results:**

Using up-to-date imaging methods, we here present morphological details of the mouthparts and the thoracopods of 168 million years old specimens of *Urda rostrata*. Mouthparts are of a sucking-piercing-type morphology, similar to the mouthparts of representatives of ectoparasitic isopods in groups such as Aegidae or Cymothoidae. The thoracopods bear strong, curved dactyli most likely for attaching to a host. Therefore, mouthpart and thoracopod morphology indicate a parasitic lifestyle of *Urda rostrata*. Based on morphological details, *Urda* seems deeply nested within the parasitic isopods of the group Cymothoida.

**Conclusions:**

Similarities to Aegidae and Cymothoidae are interpreted as ancestral characters; *Urda* is more closely related to Gnathiidae, which is therefore also interpreted as an ingroup of Cymothoida. With this position *Urda* provides crucial information for our understanding of the evolution of parasitism within isopods. Finally, the specimens reported herein represent the oldest parasitic isopods known to date.

## Background

Parasitism is a widespread strategy among animals (Metazoa), if not the most widespread one. Most, if not all parasites originated from free-living relatives. Still our understanding of how the evolution of a parasitic lifestyle evolved is not fully understood. It has been suggested that there are morphological, physiological, or ecological pre-adaptations to parasitism [1–4].

For improving our understanding of the evolution of parasitism, insects have been considered to be an especially interesting group. It seems that in various insect lineages clear pre-adaptations, such as elongated mouthparts, can be identified [5]. One model example for studying evolution of parasitism and co-evolution between the parasite and host are lice, possibly due to human medical health and livestock health interest. Chewing lice (‘Mallophaga’), specialized for a parasitic lifestyle on birds [6], have been proposed to have evolved from a free-living relative [7].

Comparable to lice, an evolutionary origin from free-living relatives, has been reconstructed for other parasitic groups, for example several worms, such as parasitic nematode worms [2, 8], parasitic flatworms [9], acanthocephalan worms [10], but also other groups closer related to mallophagan lice, such as mites [11], or parasitic isopod crustaceans [12].

Isopod crustaceans – woodlice, slaters, pill bugs and their relatives – are very diverse and successful malacostracan crustaceans (the group containing e.g. crabs, lobsters, shrimps, krill and crayfish). Isopods inhabit various habitats, including marine, freshwater and terrestrial environments [12–18]. They have developed various kinds of lifestyles, among them free-living [19], scavenging [20–22] or predatory [23], but also parasitic forms of varying degrees of specialization [24–27]. This is nicely exemplified by the isopod ingroup Cymothoida sensu Wägele [12]. Within this group numerous lifestyles have evolved, some quite soon after the appearance of the group [28]. Also, as isopods have potential to be preserved as fossils this group allows a degree of estimation of the appearance of such strategies within Earth history:

- A scavenging lifestyle is known from representatives of Cirolanidae. Fossil representatives of this group, indirectly suggesting a similar lifestyle, have been reported from the Jurassic [21] and Cretaceous [22]. Representatives of Corallanidae and Aegidae have a lifestyle reminiscent of that of a mosquito; one may interpret this as quasi-predatory behavior, yet more precisely it is a temporary parasitic lifestyle; they attach briefly to a host, a fish, only during feeding. An aegiid fossil [29] has been reported from the Late Miocene, indicating a similar lifestyle at this time. Phylogenetic inference would suggest an older origin of a “marine mosquito” strategy.
- Representatives of Cymothoidae feed similarly to aegiid isopods when they are juveniles. Yet, as adults they attach to a host fish permanently. The oldest fossil indicating such a type of parasitism in Cymothoidae has been reported from the Jurassic [30].
- During a specific larval phase, representatives of Gnathiidae feed in a comparable way to representatives of adult Aegidae and juvenile Cymothidae [20, 31]. Yet, as adults gnathiid isopods are not parasitic. An ingroup position of gnathiids within Cymothoida is equivocal ([32] vs. [33]). So far no fossils of this lineage have been reported.
- A host change respective to their ontogenetic phase can be observed also in representatives of Epicaridea. Larval epicaridids parasitize small crustaceans, e.g. copepods. Adult epicaridids infest mainly larger crustaceans, some are even quasi-endoparasitic. Based on malformations on the host [24, 27] or by comparing the life habits of modern relative groups [34], this lifestyle must have been present since the Jurassic.

These examples illustrate not only the diversity of life styles within Cymothoida. They also illustrate different ways of inferring a specific lifestyle in fossils [5]: 1) The most direct case is finding a parasite directly associated with a host [30]. 2) A more indirect way is finding isolated specimens with specific morphologies [34]. More indirect cases are (3) findings of developmental stages with a different lifestyle [35] and (4) teratological changes in the morphology of a host [24].

For 2) functional morphology and comparison to extant relatives can support interpretations of different lifestyles. Hook-like claws at the end of thoracopods for attachment in an isopod give a clear hint to a parasitic lifestyle in contrast to small, straight and pointed tips that could be used for walking locomotion.

Similarly, also for phylogenetic interpretations of fossils morphological characters, such as details of appendages on the head and thorax are crucial [12, 28, 32]. Currently most fossil isopods are mainly interpreted based on dorsal characters, as ventral morphological characters of most fossil isopods are not accessible [29, 36–38].

Yet, under certain preservation conditions more or less complete fossil isopods can be recovered, preserving ventral details, such as appendages and even appendage sub-structures, such as spines and setae. Numerous such well-preserved fossil isopods have been reported from the Mesozoic, especially from Jurassic Konservat Lagerstätten with exceptional preservation [22, 36, 39–44].

One group of isopods that is regularly found in the Jurassic is *Urda*. This genus currently includes eight species (see Table 1). So far it has neither been possible to reliably interpret the phylogenetic position of *Urda* nor its lifestyle as descriptions concentrated on dorsal characters. Yet, some authors have suggested a closer relationship of *Urda* to parasitic isopod groups, such as Aegidae, Cymothoidae or Gnathiidae (see discussion).

**Table 1:** Summary of *Urda* spp. occurrences in literature and their preservation

Current taxonomic assignment	Original taxonomic assignment	Age (stage and range in mya) after [92]	Country	Reference(s), original reference indicated by	Preservation group (1 = entire animal, 2 = anterior portion only, 3 = posterior portion only)
*U. cretacea*	*U. cretacea* [45]	Berriasian, 140-145	Germany	[45]	3 (tIII-plV)
*U. cretacea*	*U. cretacea* [45]	Berriasian, 140-145	Germany	[45]	2 (ct-tIV)
*U. cretacea*	*U. cretacea* [45]	Berriasian, 140-145	Germany	[45, 46]	1
*U.* cf. *cretacea*	*U. cretacea* [47]	Aptian, 113-125	Antarctica	[47]	3 (plI-pt)
*U. liasica*	*U. liasica* [48]	Toarcian, 174-182	Germany	[48]	3 (tVI-pt)
*U. mccoyi*	*Palaega mccoyi* [49]	Oxfordian, 157-163	Scotland	[37]	1
*U. moravica*	*U. moravica* [50]	Bathonian, 166-168	Bohemia	[50, 46]	3 (tVI-pt)
*U. punctata*	*U. punctata* [51]	Tithonian, 145-152	Germany	[51, 45, 46, 52, 53]	1
*U. rhodanica*	*U. rhodanica* [46]	Callovian, 163-166	France	[46]	3 (tV-pt)
*U. rostrata.*	*Urda* sp.[54, 55]	Bathonian, 166-168	Germany	this study	1
*U. rostrata.*	*Urda* sp.[54, 55]	Bathonian, 166-168	Germany	this study	2 (ct-tIII)
*U. rostrata*	*U. rostrata* [51]	Tithonian, 145-152	Germany	[51, 45, 46, 52, 53, 56, 57]	2 (ct-tVIII)
*U. rostrata*	*U. “cincta” *[57]	Tithonian, 145-152	Germany	[52]	1
*U. rostrata*	*U.* “*cincta” *[51, 57]	Tithonian, 145-152	Germany	[52]	1
*U. rostrata*	*U. “elongata” *[51]*,* *U. “cincta” *[57]	Tithonian, 145-152	Germany	[52]	1
*U. rostrata*	*U. “decorata” *[51]*,* *U. “cincta” *[57]	Tithonian, 145-152	Germany	[52]	1
*U. zelandica*	*U. zelandica* [56]	Tithonian, 145-152	New Zealand	[56]	3 (tVI-pt)
*Urda* sp.	*Urda* sp. [48]	Pliensbachian, 183-191	Germany	[48]	2 (ct-tIII)
*Urda* sp.	*Urda* sp. [58]	Aalenian, 170-174	Switzerland	[58]	3 (tVI-pt)

Here we present two specimens of *Urda rostrata* from the Bathonian (168 mya) of Bethel-Bielefeld (Germany). Specimens were documented with the aid of micro CT and reveal crucial characters indicating that these fossils represent the oldest fossil parasitic isopod known to date. With this they contribute novel information to the evolution of parasitism within Cymothoida.

## Methods

### Material

**Fig. 1:**
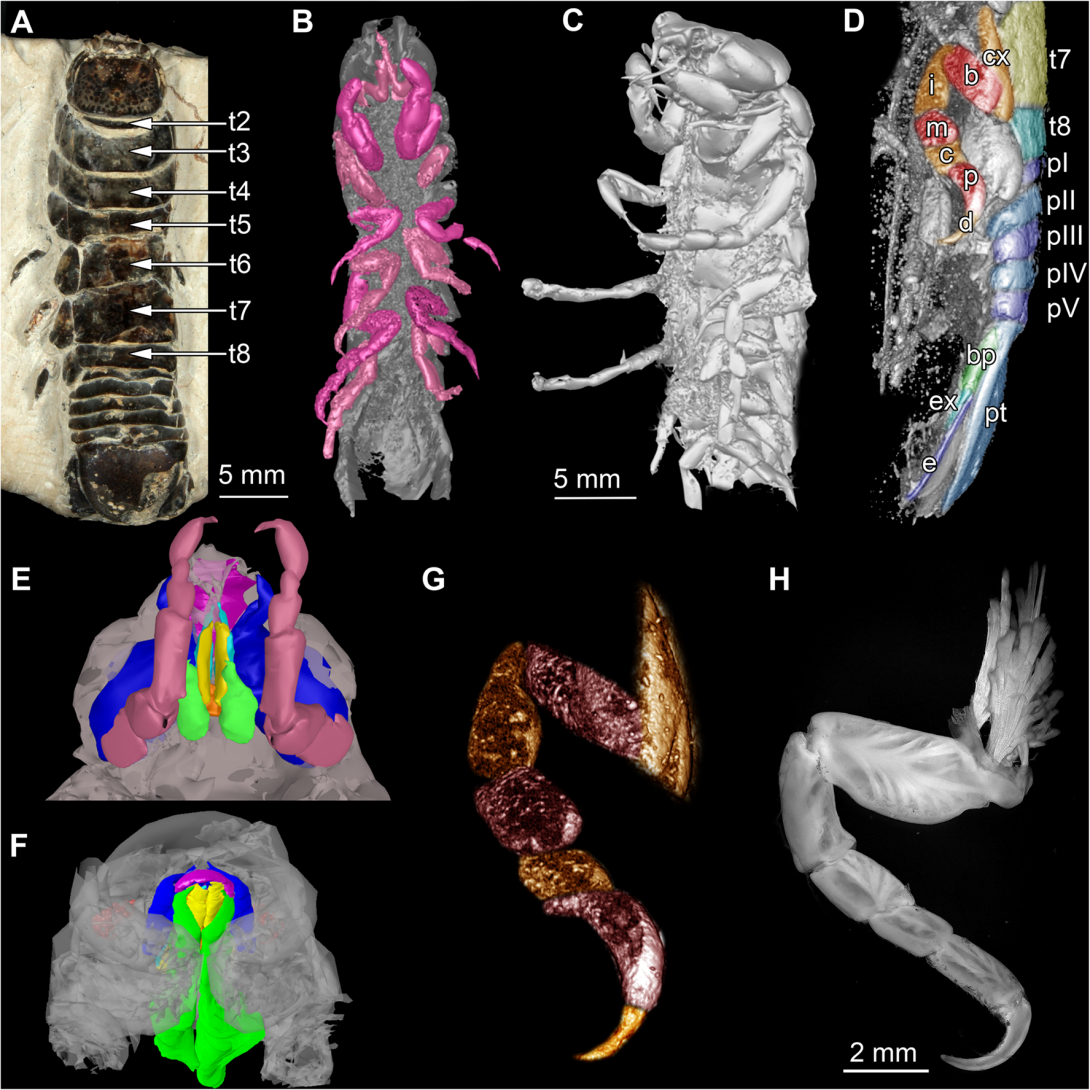
Fossil specimens and modern counterparts of isopod crustaceans. *Urda rostrata:* specimen 1 (BPSG 2011I50; **a**-**b**, **d**, **g**); specimen 2 (BPSG 2011I51; **c**, **e**); *Nerocila bivitatta* (**f**); *Anilocra physodes* (**h**). Color-marks: labrum = purple, mandibles = blue, paragnaths = orange, maxillulae = cyan, maxillae = yellow, maxillipeds = green, thoracopods = pink, thoracopod elements = red and orange, pleon segments = blue and light blue. **a** Macro photograph with indicated free thorax segments (t2-t8), dorsal view. **b**-**g** Reconstructed surface models. **b** Specimen 1 with colored thoracopods, ventral view. **c** Specimen 2, ventro-lateral view. **d** Specimen 1 with colored elements of sixth free thoracopod and segments of thorax, pleon and pleotelson. **e** Specimen 1, ventral view. **f** Functional head. **g** Sixth thoracopod of specimen 1. **h** Fluorescence microscopic photography of sixth thoracopod
- Specimen 2 (BPSG 2011I51, Figs. 1c and 3d-f) is 20 mm long from the anterior end of the cephalothorax to seventh thorax segment. Pleotelson is missing.

We investigated two fossil isopod specimens, both interpreted as representatives of *Urda rostrata*. Both specimens are preserved in an ironstone-geode and come from Bethel-Bielefeld (Germany). They are therefore interpreted as being of Bathonian age, Middle Jurassic, about 168 million years old. Both specimens were found by K. Lenzer in July 1970 and first reported by Büchner [54];

- Specimen 1 (BSPG 2011I50, Figs. 1a-b, d, 3a-c and 6a-e) is 32 mm long from the anterior end of the functional head to the posterior end of the telson and 8 mm wide.

Additionally two extant specimens of parasitic isopods (Cymothoidae) were used for comparison. For comparisons of the mouthparts a female of *Nerocila acuminata* (ZSMA 20159001, Figs. 1f and 4d-e) was used. It originates from Cross Bay, Rovinj, Croatia (45°7.06’N 13°3.99’E), and is still attached to the caudal fin of a representative of Mugilidae, identified and found by R. Melzer in 2014. Preparation, documentation and methodological proceedings have been described in Nagler and Haug [59]. For comparison of the thoracopods a female of *Anilocra physodes* (ZSMA 04con034, Fig. 1h) was used. It was collected in the Atlantic (21°19.5’N, 17°13.1’W) by L. Tiefenbacher in 1975.

### Documentation methods

Specimens were investigated with macro-photography and x-ray micro-CT scanning.

Macro-photography, combined with composite imaging (stacks of images of several adjacent image details) was performed following e.g. [60–62] under cross-polarized light. We used a Canon EOS Rebel T3i camera, either with a Canon EFS (18-55 mm) lens (for overview images) or a Canon MP-E (65 mm) macro lens (for close-up images). Illumination was provided by a Canon Macro Twin Lite MT-24EX flash from the two opposing sides to provide even illumination.

Fluorescence microscopy of the sixth thoracopods of *A. physodes* was performed on an inverse fluorescence microscope BZ-9000 (BIOREVO, Keyence) with a DAPI filter (λ = 358-461 nm) recording auto fluorescence and 10x objective resulting in about 100x magnification. Several focus layers (stacks of images) were recorded.

Stacks of images were processed with the freeware packages CombineZP (Alan Hadley), ImageAnalyzer (Meesoft) and ImageJ (Wayne Rasband). Assembling of stereo images and final processing (levels, sharpness, and saturation) was performed in Adobe Photoshop CS4.

Micro-CT scanning was performed on a Nanotom m Phoenix (GE Sensing & Inspection Technologies GmbH). An overview scan of specimen 1 ran 60 min with 140 kV and 60 μA, resulting in a calculated voxel size of 15.8 μm^3^. For specimen 2, the scan took 53 min with 140 kV and 60 mA, resulting in a calculated voxel size of 16.6 μm^3^. Scans were reconstructed to tiff stacks with the built-in software. Tiff stacks were further processed with ImageJ and Osirix 5.8.2 (Antoine Rosset). Surface models and volume renderings of both specimens, of thoracopods of specimen 1 and of mouthparts of specimens 1 and 2 were created (“segmented” or by thresholds) in Osirix. The surface models were further modified and rendered with Blender 2.49 (Blender Foundation). Stereo volume renderings and surface models of specimen 1 and specimen 2 were created (“segmented” or by thresholds) in Osirix. Surface models were further modified and rendered with Blender 2.49 (Blender Foundation).

### Presentation method

The description is focused on preserved structures that give information about the lifestyle of these isopods, i.e. functional morphology of the mouthparts and thoracopods, as these appendages are in direct contact with the host. For a better recognition we present colour-marked images of the important appendages.

### Terminology

Due to the necessity for a uniform terminology among arthropod workers [63], we choose expressions that allow an unambiguous connection of term and structure. Therefore, we use thoracopod II-VIII instead of ‘pereiopod 1-7’ (or ‘peraeopod 1-7’). We also avoid terminology implying serial homology of structures that have independent evolutionary origins; hence we use maxillula and maxilla (instead of maxilla one and two).

## Results

Description of the two fossils (specimen 1 BSPG 2011I50; specimen 2 BSPG 2011I51) is focused on preserved structures that give information about the lifestyle of the two isopod crustaceans. Therefore especially the morphology of mouthparts and thoracopods is the focus of the description, as these appendages are in direct contact with the host.

**Fig. 2:**
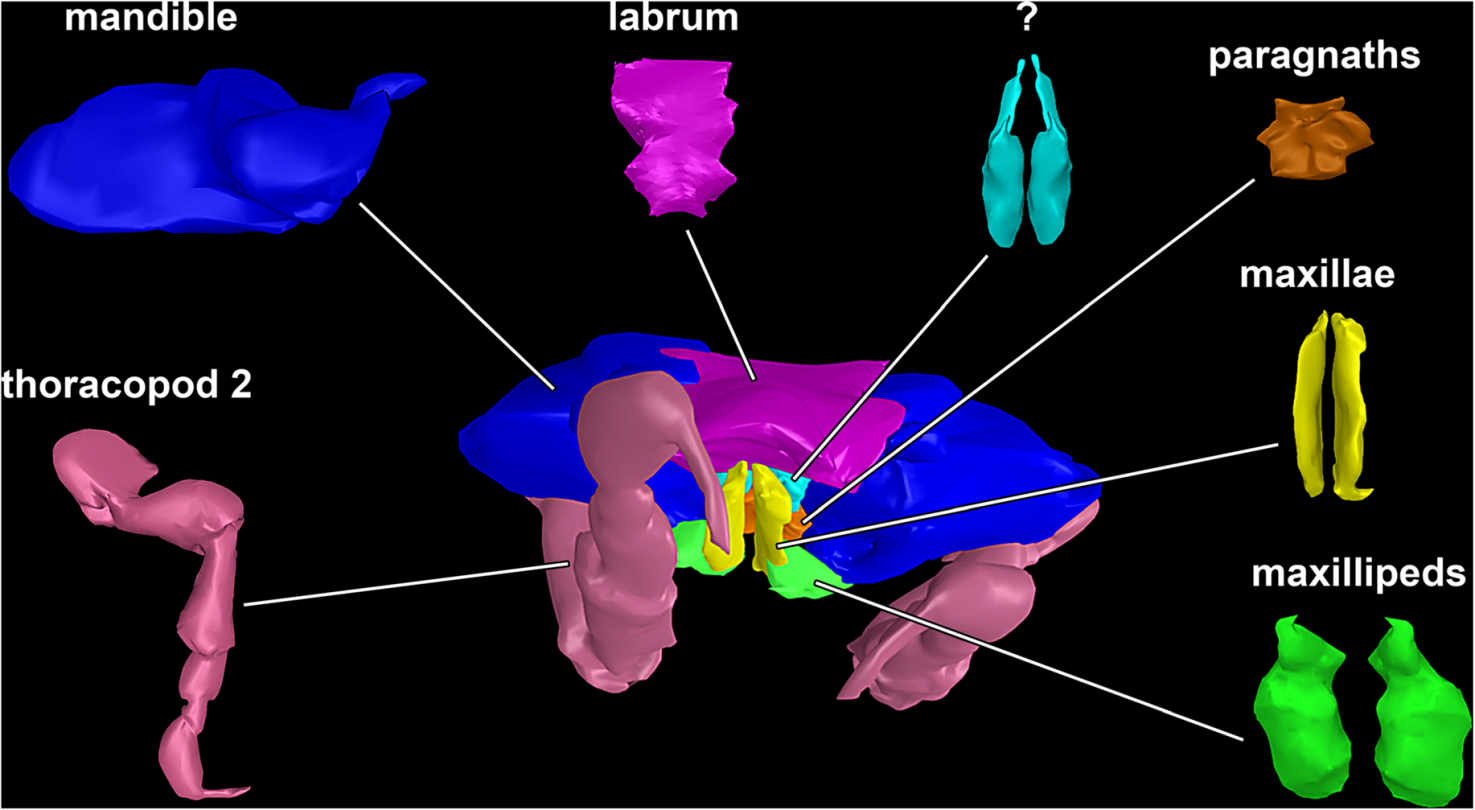
Reconstructed surface model of the mouthparts of the fossil isopod *Urda rostrata* (BSPG 2011I51). Mouthparts together in anterior view, individual mouthparts in dorsal view. Mouthpart of unclear identity (?) may either represent the maxillula or the distal region of the paragnaths. Not to scale

**Fig. 3:**
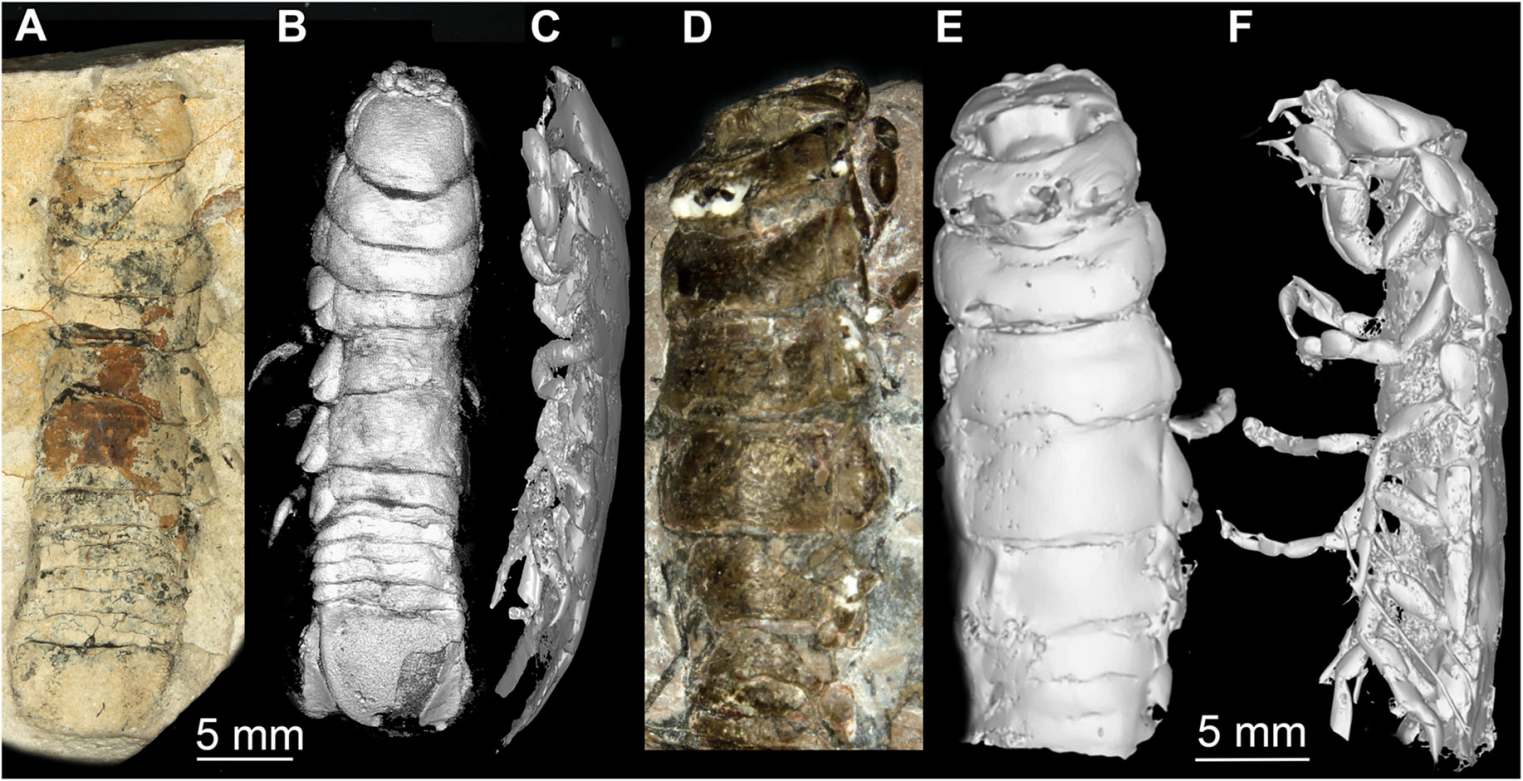
Macro photographs (**a**, **d**), volume renderings (**b**, **e**) and reconstructed surface models (**c**, **f**) of fossil isopod *Urda rostrata*. **a**-**c**) *U. rostrata* specimen 1 (BPSG 2011I50). **a** Counterpart, dorsal view. **b** Dorsal view. **c** Lateral view. **d**-**f** *U. rostrata* specimen 2 (BPSG 2011I51). **d** Latero-dorsal view. **e** Dorsal view. **f** Lateral view

**Fig. 4:**
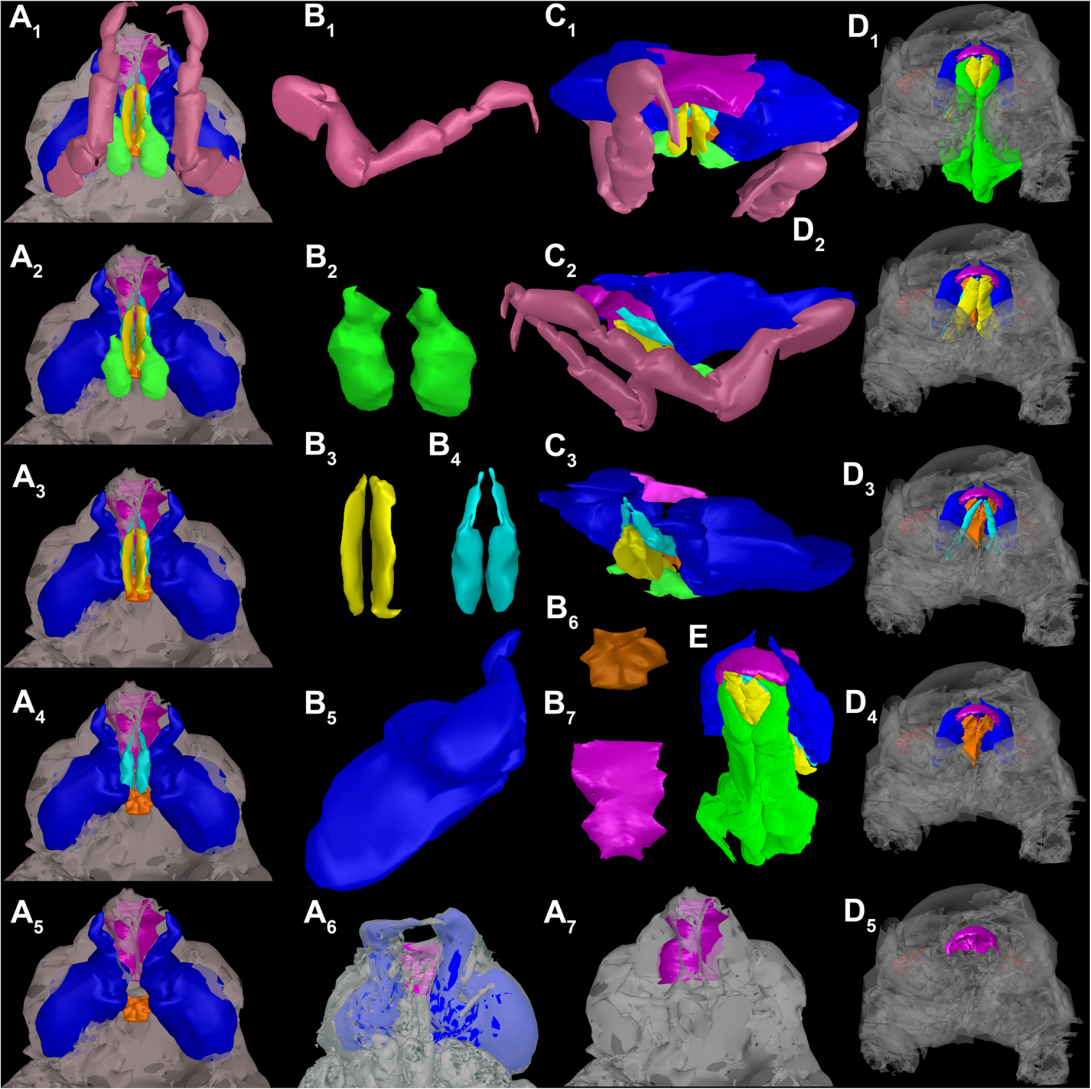
Reconstructed surface models of the fossil isopod *Urda rostrata* and modern isopod *Nerocila acuminata*. Abbreviations: tp = first thoracopods, mxp = maxillipeds, mxa = maxillae, uc = unclear mouth part, md = mandibles, pg = paragnaths, lb = labrum. **a**_1-5, 7_ Functional head of *U. rostrata* specimen 2 (BPSG 2011I51) with the first free thorax segment, successively one appendage removed from posterior to anterior, ventral view. **a**_6_ Functional head of *U. rostrata* specimen 1 (BPSG 2011I50). **b**_1-7_ Mouthparts of *U. rostrata* specimen 2. **b**_1_) Tp. **b**_2_) Mxp. **b**_3_) Mxa. **b**_4_) Uc. **b**_5_) Md. **b**_6_) Pg. **b**_7_) Lb. **c**_1-3_ Mouthparts of *U. rostrata* specimen 2, from different angles. **d**_1-5_) Functional head of *N. acuminata*, successively one appendage removed from posterior to anterior, ventral view. Not to scale

Specimen 1 is more or less complete (Figs. 1a-b, d, g, 3a-c, 4a_6_, 5a-b, d-e and 6a-e); specimen 2 is only preserved anteriorly including the first pleon segment; further posterior structures are missing (Figs. 1c, e, 2 and 3d-f, 4a_1-5, 7_, b-c and 5c).

**Fig. 5:**
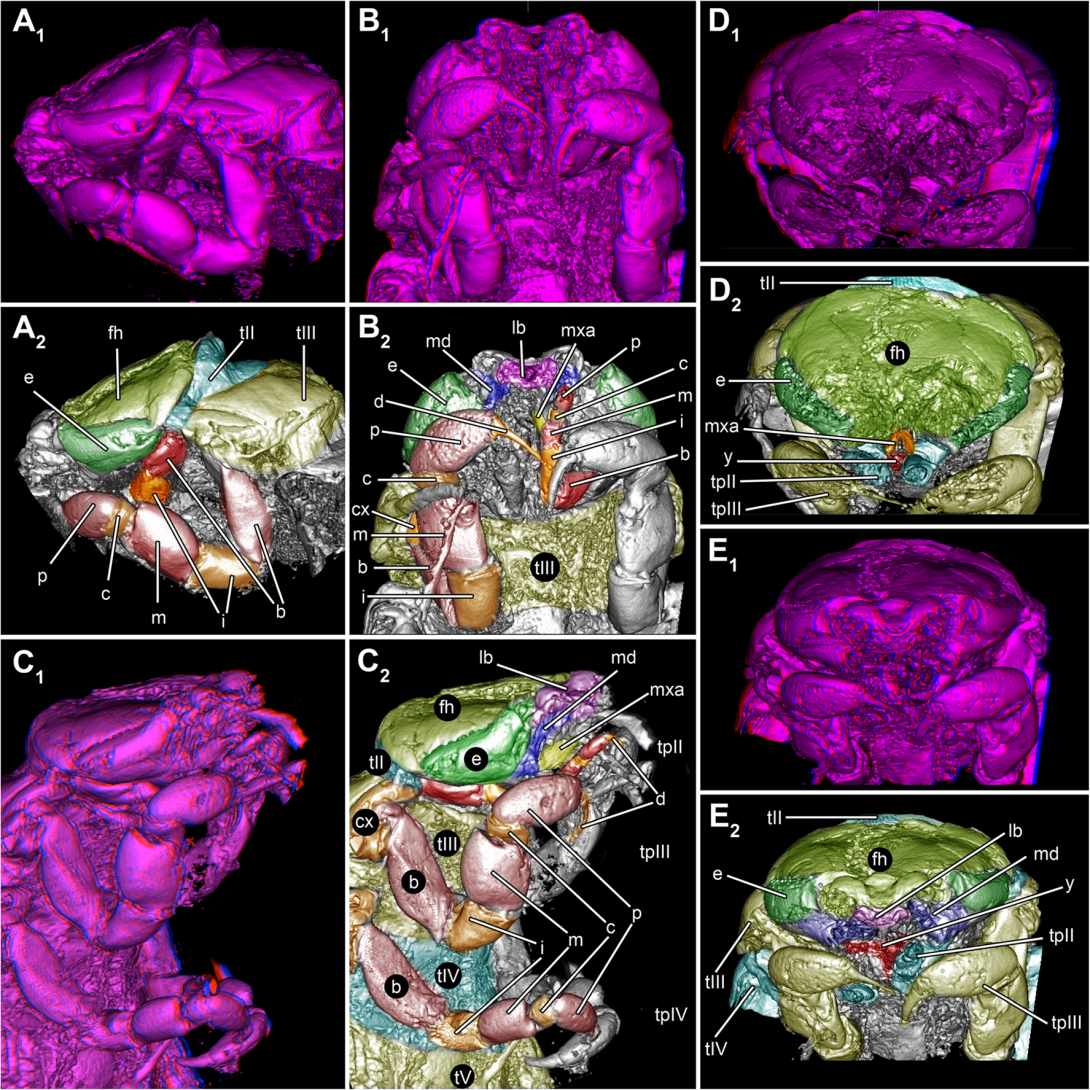
Stereo images and respectively colour marked versions of volume rendering of the functional head and the anterior region of the thorax of fossil isopod *Urda rostrata.* Abbreviations: fh = functional head, e = eye, lb = labrum, md = mandible, mxa = maxillae, y = y-shaped mouth opening, cx = coxal plate, b = basis, i = ischium, m = merus, c = carpus, p = propodus, d = dactylus, tII-V = thorax segments II-V, tpII-IV = thoracopods II-IV. **a**-**d** Specimen 1 (BPSG 2011I50). **a**1-2 Lateral view. **b**1-2 Ventral view. **c**1-2 Specimen 2 (BPSG 2011I51), lateral view. **d**-**e** Specimen 1 (BPSG 2011I50). **d**1-2 Anterior view. **e**1-2 Anterior-ventral view. Not to scale

### Body organization

**Fig. 6:**
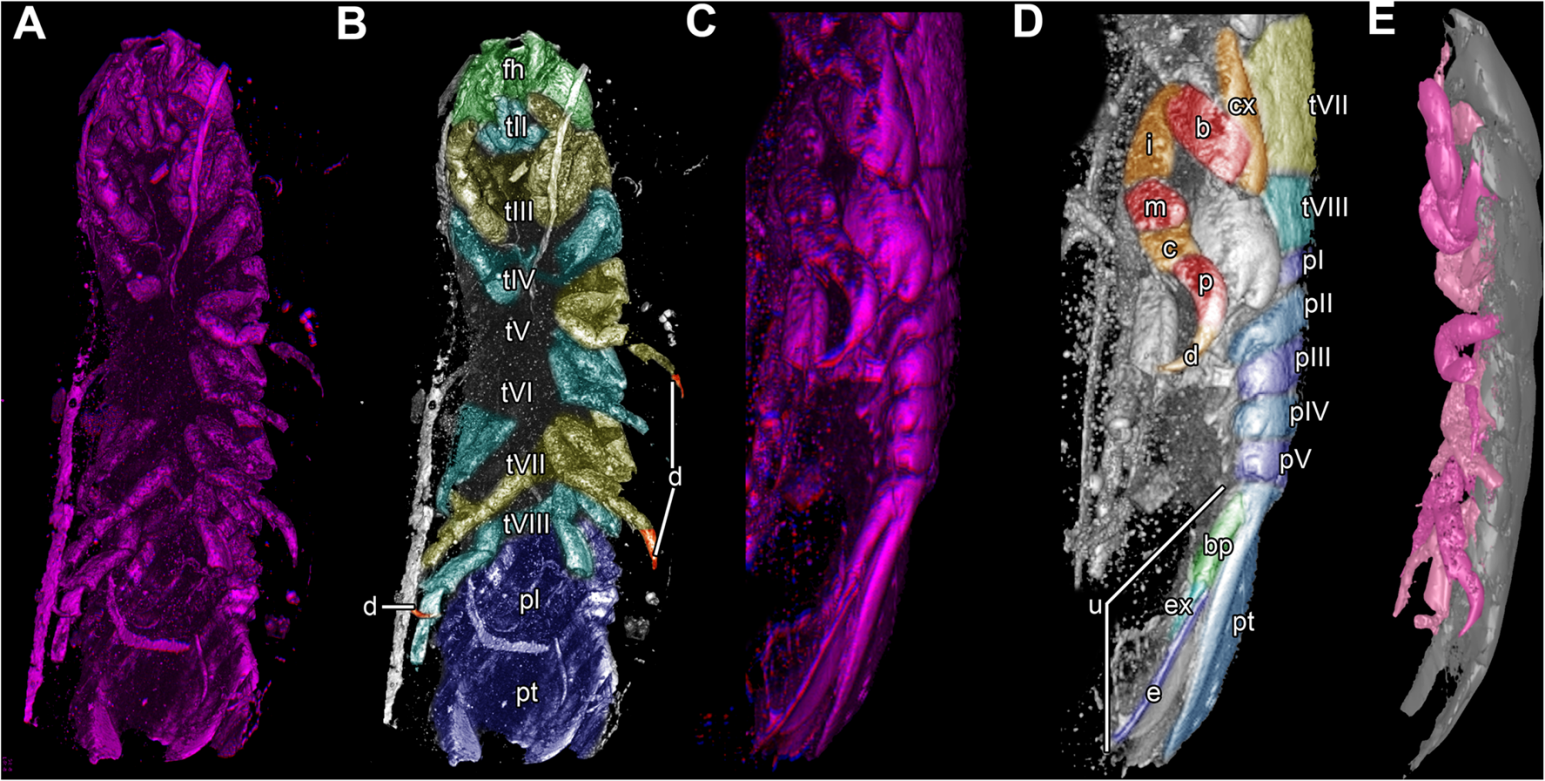
Stereo images, respectively colour marked versions of volume rendering of the thoracopods of fossil isopod *Urda rostrata* specimen 1 (BPSG 2011I50). Abbreviations: fh = functional head, tII-VIII = thoraxsegments II-VIII, cx = coxal plate, b = basis, i = ischium, m = merus, c = carpus, p = propodus, d = dactylus, pI-V = pleon segments I-V, pt = pleotelson, bp = basipod, ex = exopod, en = endopod, u = uropod. **a**-**b** Ventral view. **c**-**e** Lateral view. Not to scale

Both specimens three-dimensionally preserved (Figs. 1b-c, 3a-f and 6a-e). In visible sclerotised body areas cuticle appears tuberculate. Entire body elongate slightly dorso-ventrally flattened, with more or less constant width, but tapering anteriorly and posteriorly.

Body organized into functional head (cephalothorax; seven segments), posterior thorax (pereon; seven free segments) and pleon (five free pleon segments and pleotelson, conjoined structure of pleon segment six and telson).

Functional head consists of a eucrustacean head (ocular segment plus five appendage-bearing segments) and first original thorax segment (Fig. 1e). Head segments form dorsally a single capsulate shield. Head shield sub-rectangular in dorsal view (Fig. 5d). Head segments bear mouthparts ventrally. Each free thorax segment, dorsally forms a sclerotisation, tergite and bears a pair of appendages ventrally (Fig. 5). Tergite of first free thorax segment (thorax segment II) much smaller than those of following segments; not extending as far laterally as functional head or other tergites; very short in anterior-posterior dimensions. Tergite of second free thorax segment (thorax segment III) shows slight anterior indentation medially to match up with smaller anterior tergite. Five pleon segments, each of them roughly as long as one third of the most posterior free thorax segment. Pleotelson shape partly unclear, with a rounded posterior edge in dorsal view; slightly wider than pleon segments (Fig. 6).

### Structures of the anterior body

Description of structures of functional head is largely based on morphology of specimen 2.

Large lateral compound eyes (Figs. 1a, c and 5a-e) situated antero-laterally on functional head; reniform outline. Appendages of post-ocular segments 1 and 2 (antennula and antenna) are not preserved.

Mouthparts (appendages of ocular segment and posterior head appendages) together forming truncated cone (Figs. 1e, 4c and 5a-e); labrum confines the cone from anterior, maxillipeds and thoracopods II seal cone from the posterior. Oral opening is Y-shaped (Fig. 5e).

Labrum (upper lip, medially fused appendages of ocular segment) elongated; projecting forward from the functional head (Figs. 2, 4a, b_7_, c and 5b-c, e); crescent-shaped in distal view (doming anteriorly; functionally dorsally), trapezoid in dorsal view; partly covering remaining mouthparts from anterior.

Appendages of post-ocular segment 3 (mandible) with prominent and stout proximal part (coxa; Figs 2, 4a_1-6_, b_5_, c and 5b-c, e). Coxa rounded rectangular in anterior view. Disto-medially coxa is drawn out into process with massive sharp tip (incisor) at distal end. Distal part (mandibular palp) not preserved (unclear if original condition).

Paired protrusions of the mandibular sternum, paragnaths appear partly preserved (Figs. 2 and 4a_1-5_, b_6_, c); medially conjoined. More or less square-shaped in ventral view, with pointed projections in the middle. Medially forming a funnel, due to a depression (Figs. 2 and 4b_6_).

Paired mouthpart of unclear identity slender and elongated with two possible elements or region (large tube-shaped proximal element and thinner flattened distal element); each ending in pointed tip (Figs. 2 and 4a_1-4_, b_4_, c). Mouthpart is protruding further distally than any other mouthpart. Mouthpart may represent the distal part of the paragnaths or the appendages of post-ocular segment 4 (maxillula).

Appendages of post-ocular segment 5 (maxilla) elongated, chisel-like, (3.5 times as long as broad), flattened in anterior-posterior axis (Figs. 2, 4a_1-3_, b_3_, c and 5b-d), distal end with three spines.

Appendages of post-ocular segment 6 (maxilliped, thoracopod I), broad and stout with two elements (Figs. 1e, 2 and 4a_1-2_, b_2_, c); distal part bears prominent hook-like spine with a sharp, pointed, recurved tip.

Appendages of post-ocular segment 7 (thoracopod II) with seven elements along main axis. Element 1 (coxa), prominent, stout. Element 2 (basipod) longer, widening distally. Element 3 (ischium) as long as element 2, also widening distally. Element 4 (merus) shorter (50%), tube-shaped. Element 5 (carpus) shorter (50%). Element 6 (propodus) swollen, as long as two third of basipod. Element 7 (dactylus) recurved, hook-like (Figs. 2, 4a_1_, b_1_, c_1-2_ and 5a-e).

### Appendages on posterior thorax and pleon

Description of appendages of post-ocular segment 8-9 (thoracopods II-III) mainly based on morphology of specimen 2 (Figs. 1e, 2, 4a_1_, b_1_, c_1-2_ and 5c), description of appendages of post-ocular segment 10-13 (thoracopods IV-VIII) and post-ocular segment 19 (uropods) based on morphology of specimen 1 (Figs. 1d and 6a-e).

Appendages of post-ocular segment 8-13 (thoracopods II-VIII) each consist of seven elements (similar to thoracopod I); all about twice the size of thoracopod I, largely similar in organization to thoracopod I. All elements 7 (dactyli) at least as long as elements 6 (propodus) (Figs. 1g and 6a-d); elongated, strongly curved, hook-like (Figs. 1g, 4b_1_, 5a-e and 6a-d).

Thoracopods II-IV rotated roughly 30° degrees forward, resulting in elements 4-6 (merus, carpus and propodus) being directed diagonally towards the anterior outer edge of body. Thoracopods V-VIII rotated roughly 30° degrees backward resulting in elements 4-6 (merus, carpus, propodus) being directed diagonally towards the posterior outer edge of body. Dactyli of thoracopods III-IV curved in a ventro-median direction (Figs. 1b, 5c and 6a-d), dactyli of thoracopods V-VIII more inclined backwards (Figs. 1b and 6a-d).

Appendages of post-ocular segment 14-18 (pleopods I-V) not preserved (Figs. 1b and 3a-f). Appendages of post-ocular segment 14-19 (uropods) with basipod carrying two distal rami, endopod and exopod; both similar sized. Uropod forming tail fan with pleotelson (Figs. 1d and 6c-e).

## Discussion

### Inferring the lifestyle

The lifestyle of representatives of *Urda* has so far largely been discussed in an anecdotal way. This led, for example, to interpretations of these isopods as scavengers due to a proposed position within Cirolanidae [58] or a swimming lifestyle [64]. Yet, alternative interpretations have also been forward, for example a supposed closer relationship to Gnathiidae [46], Aegidae [39] or Cymothoidae [52, 65]. Such phylogenetic interpretations would indicate an at least partly parasitic lifestyle for representatives of *Urda*. Support for such interpretations has been largely lacking, as the critical morphological characters, such as the mouthparts and thoracopods, were not visible or only partly preserved [45–47, 58]. With our finding we can now contribute to this aspect.

#### Mouthparts

Modern parasitic isopods (especially within Cymothoida) in general have some of their mouthparts elongated, rotated on their axis distally (45° from the horizontal axis of the animal), and together forming a more or less tight sucking and/or piercing mouth cone, (Fig. 1f) [59, 66]. Such an arrangement also appears to be present in the fossil specimens studied here (Figs. 1e, 2, 4c-d and 5a-e).

The forward projecting labrum of the here presented fossils (Figs. 2 and 4a_6-7_, b_7_, c) is similar in shape and position to that of representatives of modern parasitic isopods of the groups Aegidae and Cymothoidae (Fig. 5d-e). In representatives of these groups this type of labrum prohibits loss of fluids when feeding on the host by sealing the mouth cone from the anterior [59, 67].

The maxilla and the second mouthpart of unclear identity in the fossil are very elongated and rotated off axis (Fig. 2). Such a type of maxilla is known in representatives of Aegidae and Cymothoidae and act as piercing structures (Fig. 4d_3_).

The second mouthpart in the fossil is more difficult to interpret as, based on its position, it may represent either the maxillula or parts of the paragnaths. In representatives of Aegidae and Cymothoidae the maxillula is also elongated, as it is in larval, parasitic forms of Gnathiidae [12, 68–70]. The structure seen in the fossil could thus be interpreted as the maxillula. Yet, in adult representatives of Gnathiidae the maxillula is absent and the distal parts of the paragnaths are comparably elongated. This is therefore also a possible interpretation for the fossils.

While the arrangement of the fossils’ mouthparts clearly shows that these possess a mouth cone, it differs from that of representatives of Aegidae and Cymothoidae (Figs. 1f and 4d-e). The mouthparts of the fossils appear to form a more loose type of mouth cone (Figs. 1e, 2, 4c and 5a-e). This more loose appearance is caused by 1) the absence of a mandibular palp (Figs. 2 and 4b_5_) that in representatives of Cymothoidae “grasps” around the labrum further sealing it [59], and 2) the relatively smaller maxillipeds (Fig. 4). The arrangement is therefore more comparable to that in larval representatives of Gnathiidae, where the mouthparts also only form a very loose type of cone [12, 65]. In these larvae the labrum and maxillipeds leave even more areas open than in the fossil.

The mandibles of representatives of Cymothoidae have a triangular blade-like incisor region, to cut pieces of tissues off the host [59, 71, 72]. This is different to the fossils, where the pointed, hooked mandibles were most likely used for piercing movements.

A further important observation is that the first free thorax segment (second thorax segment) is partly incorporated into the functional head (Figs. 1e, 2, 4a_1_, c and 5a-e). This condition is indicated by the small size of the tergite as well as the far anterior position of the appendage, as well as its size. This appendage may have been used to grasp into the host to provide pressure when inserting the mouthparts into the host. This distantly resembles the condition in Gnathiidae, yet in representatives of this group the segment of the second thoracopod is fully integrated into the functional head [65].

We can conclude that the mouthparts of the fossils investigated here strongly resemble those of modern parasitic forms, such as Aegidae, Cymothoidae and Gnathiidae in many aspects. This makes a parasitic lifestyle, probably on a fish host, for the fossil isopods likely.

#### Thoracopods

The dactyli of all thoracopods of the fossils are strongly curved, i.e. roughly modified into a hook (Figs. 1b, g, 3 and 6). This resembles dactyli of modern parasitic isopods (Fig. 1h). In modern forms, such as representatives of Cymothoidae, such hook-like dactyli are used for attaching to the host [25, 59, 73]. Additionally in modern forms the arrangement of the dactyli is adapted for prohibiting removal from the host [59]. A very similar pattern is seen in the fossil specimens, although the first free thoracopod, tII, is partly incorporated into the head. Still, the dactyli of thoracopods II-V grasp into the host at a 90° angle to the isopod’s body, whereas the dactyli of the remaining thoracopods VI-VIII are more inclined backwards, and in this way strongly resemble modern forms [59].

To summarize, not only the morphology of the mouthparts (Figs. 1e, 2, 4 and 5), but also that of the thoracopods (Figs. 1d, g, 3 and 6) exhibit strong resemblance to similar structures in modern parasites. This similarity indicates a parasitic lifestyle of the fossil specimens studied herein.

Additional, although weaker, hints include: 1) The palaeo-environmental setting of the Bethel-Bielefeld limestone. It was interpreted as a tropical to subtropical back-reef lagoon [74]. Representatives of the obligate parasitic group Cymothoidae are nowadays most diverse in such ecosystems [32, 75]. 2) The dorso-ventrally flattened body of the fossils (Figs. 1a-d and 3a-f). This body shape is in contrast to, e.g., free-living cirolanids [76]. This can be understood as an additional adaptation for parasitism, reducing water resistance for the host. Similar adaptations are known in modern parasitic forms [77]. 3) The size of the fossils. With at least 30 mm the specimens are relatively large. Isopods parasitizing fishes have been reported to be larger than most free-living species [78] (except for deep sea forms, such as representatives of *Bathynomus*).

The eyes of the here described specimens appear well-developed. With this they give no additional indication for a reduced visual capability, as reported for permanently attached parasitic isopods [67, 73]. Together with the appendages that are clearly modified for parasitism we suggest a lifestyle similar to modern representatives of juvenile Gnathiidae. The animal would have attached to a host for a longer time (more or less permanently), but feed on the host only for a short time. With this the overall behaviour of the fossils could be compared to a mallophagan louse. Hence, the fossils are a kind of “marine mallophagan”.

### Evolution of parasitism within Cymothoida

Historically, *Urda* has been interpreted as closely related to Cirolanidae [39, 79], to Gnathiidae [46], to both of these two groups [47, 53, 80–82], or as a subgroup of Cymothoidae [52, 65].

One major challenge to resolving this issue, besides the lack of knowledge of mouthparts and thoracopods, was a dispute on the body organization of representatives of *Urda*. Available descriptions vary between five [45, 82], six [54–56, 58, 80] and seven free thorax segments [37, 46, 51, 52]. Our specimens clearly show seven free thorax segments for *Urda rostrata* (Fig. 1a). Part of the former confusion might have been caused by the rather small tergite of the first free thorax segment.

With our newly observed features we can therefore provide a phylogenetic interpretation of *Urda*. Additionally, we can provide a new reconstruction of character evolution for parasitic isopods within Cymothoida.

### Phylogenetic interpretation

Cymothoida is a large group within Isopoda, including most (if not all, see below) of the parasitic isopods. There was most likely an evolutionary switch to parasitism that was followed by a large adaptive radiation and thus diversification of the different parasitic isopod groups.

Among the parasitic cymothoidans, it seems well established that Corallanidae is the sister group to all remaining parasitic forms [12, 28, 32, 83]. Corallanidae is united with all remaining forms by the specialization of a hook-like dactylus on thorax appendage II. Such a specialization is absent in free-living closer relatives such as representatives of Cirolanidae, and therefore appears to be an autapomorphy of the group including all the parasites.

The sister group to Corallanidae is a group including Aegidae, Cymothoidae and Epicaridea. This group has been largely accepted as monophyletic. The group is characterized (autapomorphy) by not one, but three hook-like dactyli on thorax appendages II, III and IV. Additionally they share a specialization of the mouthparts, which form a mouth cone allowing piercing and sucking (partly further specialized and reduced in different epicarids).

Epicaridea and Cymothoidae both have more than three hook-like dactyli, indicating a closer relationship between the two, and together being the sister group to Aegidae. Yet, we have not mentioned Gnathiidae. This group has been “shifted around the tree” in numerous studies [12, 28, 32, 33, 73, 83]. In this sense the fossils described here are interesting as they share certain aspects of their morphology with representatives of Gnathiidae and others with representatives of Cymothoidae and Aegidae. Representatives of Gnathiidae share certain morphological aspects with Epicaridea and Cymothoidae: all have the posterior six thoracopods modified for attaching (in contrast to Aegidae, where there are only three). We therefore suggest that *Urda* and Gnathiidae are nested within the other parasitic isopods of Cymothoida.

Yet, six hook-shaped dactyli are restricted to a specific (larval) life stage in Gnathiidae. Also the exact attachment appears modified in Gnathiidae as the propodus appears to be a functional part of the “hook”. Still the non-parasitic lifestyle of other life stages and the different attachment structure can be understood as secondary modifications. Gnathiidae and Epicaridea share the absence of the maxillula [73]. We therefore interpret these two as more closely related to each other than either of these two to Cymothoidae.

*Urda* could thus either represent the sister group of (Epicaridea + Gnathiidae) or of Gnathiidae alone. Similarities of *Urda* with Aegidae and Cymothoidae would represent plesiomorphies; a less tightly packed mouth cone and possible absence of the maxillula (if the unclear mouthpart represents the paragnaths) would unite *Urda*, Gnathiidae and Epicaridea. The partial incorporation of the thorax segment II into the head could represent a synapomorphy of *Urda* and Gnathiidae.

### Character evolution

**Fig. 7:**
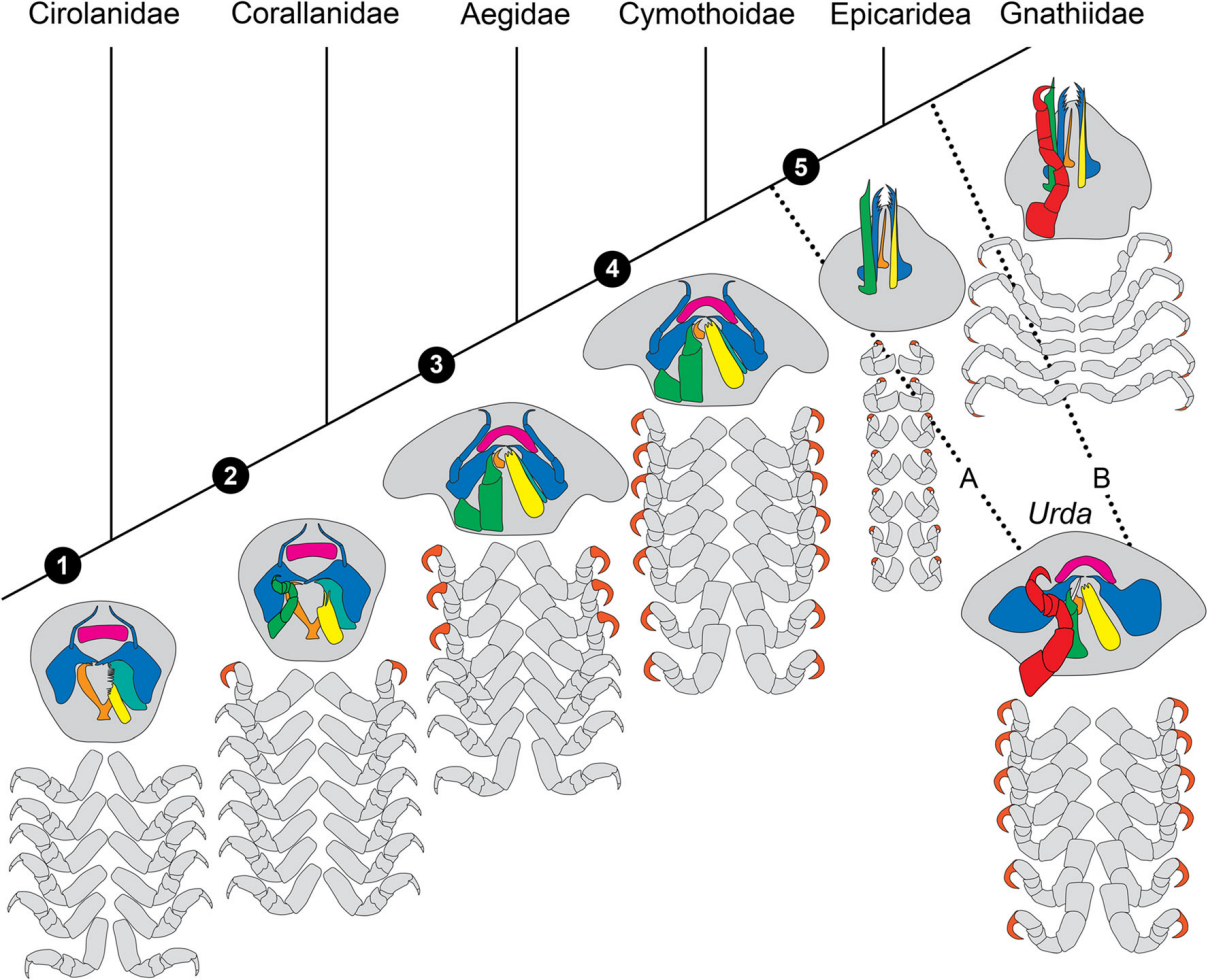
Reconstructed relationship of some groups within the isopod group Cymothoida with a schematic character evolution of the mouthparts and thoracopods. Color-marks: labrum = purple, mandibles = blue, paragnaths = orange, maxillula = cyan, unclear mouthpart = white, maxilla = yellow, maxilliped = green, first free thoracopod = red, claw-like dactyli on thoracopods = dark orange. Important steps are: 1) cirolanid-like ancestor with carnivorous mouthparts, swimming thoracopods; 2) modified mouthparts for piercing, maxilliped and first free thoracopod modified for attachment; 3) mouthparts forming a sucking mouth cone, three thoracopods modified for attachment; 4) sucking mouth cone, all seven thoracopods modified for attachment; 5) mouthparts elongated still forming a mouth cone for piercing, reduced mandibular palp and maxillula, reduced seventh thoracopod. Position of *Urda* either in position A or B (see discussion)

The proposed phylogeny would lead to a character evolution as follows (Fig. 7):

1. The ground pattern (= reconstructed morphology of the stem species) of Cymothoida (character state transition 1) includes mouthparts indicating a carnivorous mode of feeding [12, 32, 73], thoracopods are of a swimming-type [84]. The stem species was most likely a scavenger or predator on fish.
2. The ground pattern of the unnamed sister group to Cirolanidae (including Corallanidae, Aegidae, Cymothoidae, *Urda*, Gnathiidae, Epicaridea; character state transition 2) is characterized by slightly longer, thinner and more pointed mouthparts that facilitate piercing; but still a slender maxilliped. At least the second pair of thoracopods appears to have a hook-like dactylus with which they attach to fish for temporary parasitism.
3. The ground pattern of the unnamed sister group to Corallanidae (including Aegidae, Cymothoidae, *Urda*, Gnathiidae, Epicaridea; character state transition 3) is characterized by a further specialization of the mouthparts; these form a sucking mouth cone (sealed by the labrum, paragnath, maxilla, maxilliped, and maxillula and mandible are used for piercing and cutting pieces of the host) [66]. Thoracopods two to five (three pairs of thoracopods) have strong hook-like dactyli [67, 73]. Adults have retained well-developed eyes [85]. The morphology of the mouthparts and thoracopods is strongly modified for a temporary parasitism on fish, comparable to a “marine mosquito”.
4. The ground pattern of the unnamed sister group to Aegidae (including Cymothoidae, *Urda,* Gnathiidae, Epicaridea, character transition state 4) still includes a mouth cone [59, 66, 73]. Seven thoracopods (II-VIII) are now modified for better attachment to the host with hook-like dactyli [67, 73].
5. The ground pattern of the unnamed sister group of Cymothoidae (Gnathiidae, *Urda* Epicaridea, character transition state 5) is characterized by the lack of the mandibular palp and the maxillula [73]. Also the labrum is smaller and not covering the other mouthparts, resulting in a sucking and piercing mouth cone that is not as tight as in representatives of Cymothoidae of Aegidae. Thoracopods retain the hook-like dactyli.

In summary, the specialized and diverse morphology and parasitic lifestyle of representatives of Epicaridea, Gnathiidae and *Urda* originated from the scavenging life style in representatives of Cirolanidae. It occurred stepwise via a lifestyle as seen in representatives of Corallanidae, attaching to a fish and feeding on it, the still temporary parasitic lifestyle with already sucking-feeding in representatives of Aegidae and the permanent parasitic lifestyle of for example Cymothoidae. A similar evolutionary reconstruction has been proposed for other parasitic arthropods, such as lice [6]. Modern book lice, supposedly the sister group to true lice, are known to live in nests and pelage of mammals and birds and feed on remains of these larger animals [7, 86, 87]. Modern true lice, e.g. chewing and sucking lice (mallophagan and anopluran respectively) are obligatory ectoparasites on birds and mammals [88]. The ancestors of true lice had simple chewing mouthparts and were free living in the nests of vertebrates (similar to book lice). Later in their evolutionary history, they adapted from associates to parasites by feeding directly from their hosts; hence, representatives of true lice evolved more specialized mouthparts for specific hosts and consequently a large diversity of forms [5, 89]. Due to the similarity between the evolutionary reconstruction of lice and representatives of Cymothoida and the similar lifestyle of chewing lice and the fossil specimens studied herein, we refer to these fossils and similar behaving isopods as “marine mallophagans”.

## Conclusion

We provide here indirect evidence for a case of palaeoparasitology by a 168 million years old isopod. This represents the oldest possible fossil parasitic isopod to date. Furthermore, the fossils contribute important data towards the origin and diversification of parasitism within the isopod group Cymothoida. Parasitism appears to have arisen only once, further diversifying within the group. As these fossils appear deeply nested within the parasitic Cymothoida, the origin of the group and with this of a parasitic lifestyle within isopods must be even older than 168 million years.

## Abbreviations

BSPG: Bavarian state collection for palaeontology and geology
ZSM: Bavarian state collection of zoology
